# *Cirsium brevicaule* A. GRAY leaf inhibits adipogenesis in 3T3-L1 cells and C57BL/6 mice

**DOI:** 10.1186/1476-511X-12-124

**Published:** 2013-08-15

**Authors:** Masashi Inafuku, Ruwani N Nugara, Yasuo Kamiyama, Itsuki Futenma, Ayako Inafuku, Hirosuke Oku

**Affiliations:** 1 Center of Molecular Biosciences, Tropical Biosphere Research Center, University of the Ryukyus, 1 Senbaru, Nishihara, Okinawa 903-0213, Japan; 2 United Graduate School of Agricultural Sciences, Kagoshima University, 1-21-24 Korimoto, Kagoshima 890-0065, Japan; 3 Tokunoshima Tokushukai Hospital, 7588 Kametsu, Tokunoshima, Kagoshima 891-7101, Japan

**Keywords:** *Cirsium brevicaule* A. GRAY, Anti-adipogenesis, Obesity, Non-alcoholic fatty liver disease

## Abstract

**Background:**

Various flavonoids obtained from the genus *Cirsium* have been reported to exhibit beneficial effects on health. The present study evaluated the antiobesity effects of *Cirsium brevicaule* A. GRAY leaf (CL) by using 3T3-L1 cells and C57BL/6 mice that were fed a high-fat diet (HFD).

**Methods:**

Dried CL powder was serially extracted with solvents of various polarities, and these extracts were tested for antiadipogenic activity using 3T3-L1 adipocytes. Mice were fed experimental HFD supplemented with dried CL powder for 4 wk. Lipid levels and mRNA levels of genes related to lipid metabolism were determined in 3T3-L1 adipocytes and the white adipose tissue (WAT) and liver of mice fed on a HFD.

**Results:**

Treatment of 3T3-L1 adipocytes with a hexane extract of CL significantly reduced cellular lipid accumulation and expression of the fatty acid synthase (FASN) gene. Dietary CL reduced the serum levels of non-esterified fatty acids in HFD-fed mice. Significant decreases in subcutaneous WAT weight and associated FASN gene expression were observed in the mice fed the experimental CL diet. Dietary CL also reduced the hepatic lipid and serum levels of a hepatopathic indicator in the HFD-fed mice. A significant reduction in mRNA levels of FASN and HMG-CoA reductase were observed in the livers of the CL-diet group. Dietary CL, on the other hand, increased in the hepatic mRNA levels of genes related to β-oxidation, namely peroxisome proliferator-activated receptor α, calnitine palmitoyltrasferase 1A, and uncoupling protein 2. Expression of the insulin receptor gene was also significantly increased in the livers of mice-fed the CL diet.

**Conclusions:**

The present study therefore demonstrated that CL suppresses lipid accumulation in the WAT and liver partly through inhibiting mRNA levels of FASN gene and enhancing the lipolysis-related gene expression.

## Background

Obesity, a natural consequence of over-nutrition and irregular living habits, contributes to the pathogenesis of metabolic syndrome. Metabolic syndrome, which comprises a cluster of metabolic abnormalities such as hyperlipidemia, type 2 diabetes mellitus (T2DM), and hypertension, is a widespread and an increasingly prevalent disease in industrialized countries, and it has contributed to an increase in cardiovascular morbidity and mortality [1,2]. Nonalcoholic fatty liver disease (NAFLD) is also often associated with metabolic syndrome [3,4]. Several therapeutic agents have been developed for treating obesity by reducing nutrient absorption [5] or by enhancing thermogenesis and lipid turnover [5,6]. However, practical use of these drugs has been hampered by their side effects and because of the rebound weight, which is gained upon termination of these therapies. In this context, it has been considered that supplementation of the daily diet with natural anti-obesity agents would be effective for managing obesity, as well as calorie control and exercise. From viewpoint of safety, the medicinal plants and their active compounds merit investigation for development of natural antiobesity agents.

During recent decades, many studies reported the beneficial effects of flavonoids on health, and these effects were either dependent or independent of their antioxidant activities. Increasing evidence indicates that flavonoid-rich food, beverages, and extracts, as well as pure flavonoids, could ameliorate the symptoms of metabolic syndrome and its associated diseases [7]. Various flavonoids have been isolated as naturally occurring compounds from the genus *Cirsium*[8,9]. It was reported that pectolinarin and 5,7-dihydroxy-6,4’-dimethoxyflavone (DDMF) isolated from *C. japonicum* DC inhibits the growth of implanted tumors and promotes innate immunity in mice [10]. Further, these studies showed that flavonoids enhance the adipocyte differentiation and glucose uptake in 3T3-L1 cells [11] and demonstrated the antidiabetic activities of pectolinarin and DDMF in rats in which diabetes was induced by streptozotocin and a high-carbohydrate/high-fat diet [12]. The crude extracts of *C. oligophyllum* reduced the weight of subcutaneous and visceral white adipose tissue (WAT) weights in rats [13]. A significant hypoglycemic effect of *C. pascuarense* was shown in alloxan-induced diabetes [14]. *C. brevicaule* A. GRAY (CBAG), which is a wild perennial herb, grows in rocky gravels or forest margins along maritime coastlines [15], and is distributed in southern-Japan and Taiwan. Its leaves, stems and roots are traditionally used as not only a food stuff but also as herbal medicine in the Okinawa Islands and Amami Islands of Japan. However, no scientific data on the biological activities of CBAG are available. We therefore evaluated the biological activities of CBAG pertaining to metabolic syndrome. This study aimed to characterize the CBAG leaf (CL) as an antiobesity agent by testing its activities *in vitro* tissue cultures and an *in vivo* animal model.

## Materials and methods

### Preparation of CBAG leaf powder and extracts

The CBAG that was used in this study was harvested on Tokunoshima Island in Kagoshima Prefecture, Japan. The freeze-dried and ground powder of CL was generously provided by Tokunoshima-cho (Kagoshima, Japan). The CL powder was analyzed by Japan Food Research Laboratories (Tokyo, Japan) and the nutrient composition was found to be as follows: carbohydrate, 12.7%; fiber, 37.8%; mineral 19.9%; protein, 19.1%; fat, 5.3%; and moisture, 5.2%. The dried-CL powder was serially extracted by incubation with 10 volumes of hexane, chloroform, ethanol and water for 2 h at 37°C. The filtrates were evaporated or freeze-dried in vacuo, and stored at −80°C until use. The dried extracts were dissolved in dimethyl sulfoxide at 25 mg/mL and filter-sterilized before use. The total phenolic contents of the serial extracts from the CL were measured using the Folin-Denis assay method as previously reported [16]. The phenolic contents were expressed in chlorogenic acid equivalents (mg CA/g extract).

### Cell culturing and treatment

3T3-L1 preadipocytes were purchased from the American Type Culture Collection (VA, USA). 3T3-L1 cells were maintained in DMEM containing 10% calf serum at 37°C in a humidified atmosphere of 5% CO_2_. Complete confluence of the cells was avoided before initiating differentiation. For adipogenesis, confluent cells were maintained for another 2 d. And then differentiation was induced with standard differentiation procedure for 2 d in the presence of the CL extracts as mentioned previously [17]. The cells were re-fed with DMEM containing 10% FBS supplemented with 10 μg/mL human insulin and 50 μg/mL CL extract for 4 d. Cell viability was measured using MTS assay to test the cytotoxic effects of the treatment on the 3T3-L1 cells.

### Measurement of lipid accumulation in cultured adipocytes

To evaluate the effect of each CL extract on lipid accumulation, the cultured 3T3-L1 cells were stained with Oil Red O using a standard protocol, and their lipid droplets were observed under a microscope. Cellular lipid was extracted and purified by using the method of Bligh and Dyer [18], and intracellular triglyceride (TG) content was quantified using a commercial enzymatic kit as described below. The cellular protein content was determined using a Quant–iT protein assay kit (Life technologies, CA, USA).

### Animals and experimental diets

All procedures for the animal experiments were performed in conformance with the guidelines on animal experiments provided set out by the ethical committee of the Animal Welfare Center at the University of the Ryukyus. Eighteen 3-wk-old male C57BL/6 (B6) mice were purchased from Japan SLC, Inc. (Shizuoka, Japan). The mice were housed individually in plastic cages under specific pathogen-free conditions, and maintained at 24°C in a 12 h light–dark cycle. After 1 wk of adaptation, the B6 mice were randomly divided into 3 experimental HFD groups (n = 6 for each group): a control diet group, a 5% CL diet group, and a 10% CL diet group. Experimental HFD containing with 15% fat were prepared on the basis of AIN-76 formulation—its composition is summarized in Table 1. Approximately 31% of total calories were derived from fat in HFD used in this study. We prepared the HFD expeditiously in room temperature. Prepared HFD were stored at −80°C in plastic bags with nitrogen gas until use. The mice were fed the experimental HFD and water ad libitum for 4 wk. At the end of the feeding period, the mice were killed after 6 h starvation by exsanguination from the heart under anesthesia induced using pentobarbital sodium salt. Their liver and epididymal, perirenal, omental and waist subcutaneous WATs were excised and serum was separated from the blood. Liver, perirenal and subcutaneous WATs, and sera were frozen immediately in liquid nitrogen and then were stored at −80°C until use.

**Table 1:** Composition of the experimental diets

**Ingredient**	**Diet**		
	**Control**	**5% CL**	**10% CL**
		(%)	
Casein	20	19.045	18.09
β-Corn starch	15	14.365	13.73
Sucrose	39.4	38.485	37.49
Cellulose	5	3.11	1.22
Mineral mix (AIN-76)	3.5	3.5	3.5
Vitamin mix (AIN-76)	1	1	1
DL-Methionine	0.3	0.3	0.3
Choline bitartrate	0.2	0.2	0.2
Corn oil	15	14.735	14.47
Water	5.2	2.6	0.0
CL powder*	0	5	10
Total	100	100	100

*Carbohydrate, 12.7%; fiber, 37.8%; mineral 19.9%; protein, 19.1%; fat, 5.3%; moisture, 5.2%.

### Measurement of serum biochemical parameters

Serum levels of TG, total cholesterol (TC), and non-esterified fatty acids (NEFA) were determined by using a commercial enzymatic kit (Wako Pure Chemical Industries, Ltd., Osaka, Japan). The activities of aspartate aminotransferase (AST) and alanine aminotransferase (ALT) in serum were measured by using a commercially available kit (Wako Pure Chemical Industries, Ltd.) Serum levels of insulin and total adiponectin were measured by using ELISA kits that were purchased from Morinaga Institute of Biological Science, Inc. (Kanagawa, Japan) and Otsuka Pharmaceutical Co., Ltd. (Tokyo, Japan), respectively.

### Measurement of TG and TC levels in the liver

Hepatic lipids were extracted and purified according to a method reported previously [19]. Hepatic TG and TC levels were determined by using the method of Fletcher [20] and by using a commercial enzymatic kit as mentioned above, respectively.

### RNA extraction and quantitative real-time RT-PCR

Total RNA was extracted from the cultured cells or the excised tissues by using TRIzol Reagent and a PureLink RNA mini kit (Life technologies). First strand cDNA was synthesized with 2 μg of total RNA as a template. The Additional file 1: Table S1 lists the DNA sequences of specific primers that were used for quantitative real-time PCR: β-actin, 18S rRNA, mesoderm-specific transcript (MEST), peroxisome proliferator-activated receptor (PPAR) α, PPARγ, sterol regulatory element-binding protein (SREBP)-1c, SREBP-2, fatty acid binding protein 4 (FABP4), CCAAT/enhancer binding protein (C/EBP) α, fatty acid synthase (FASN), insulin receptor (IR), HMG-CoA reductase (HMGCR), calnitine palmitoyltrasferase (CPT)-1A, insulin induced gene (INSIG)-2a, acyl-CoA oxidase (AOX), and uncoupling protein (UCP)-2. The amplifications were performed in a commercial real-time PCR system (StepOne Real-Time PCR System; ABI). The relative amount of each gene transcript was normalized to the amount of β-actin transcript in adipocytes and WAT and to the amount of 18S rRNA in the liver.

### Statistical analysis

For *in vitro* experiments, the statistical significance of the difference between two experimental groups was determined by using Student’s *t*-test. The statistical significance of the differences between the mean values of the control and treatment groups were inspected by using Dunnett’s test for the animal experiments. Differences were considered significant at *P* < 0.05.

## Results

### Phenolic content of CL extracts

Serial extractions of CL powder were performed to evaluate their effectiveness *in vitro*. The yields of the dry material from extracts were as follows (g/100 g CL powder): hexane extract (1.97 g), chloroform extract (2.73 g), ethanol extract (7.33 g) and water extract (28.8 g). Of these extracts, the ethanol extract had the highest phenolic content (618 mg CA/g). The phenolic content of hexane, chloroform, and water extracts were 26.4, 70.9, and 121 mg CA/g extract, respectively.

### Effect of CL extracts on 3T3-L1 adipocytes

**Figure 1:**
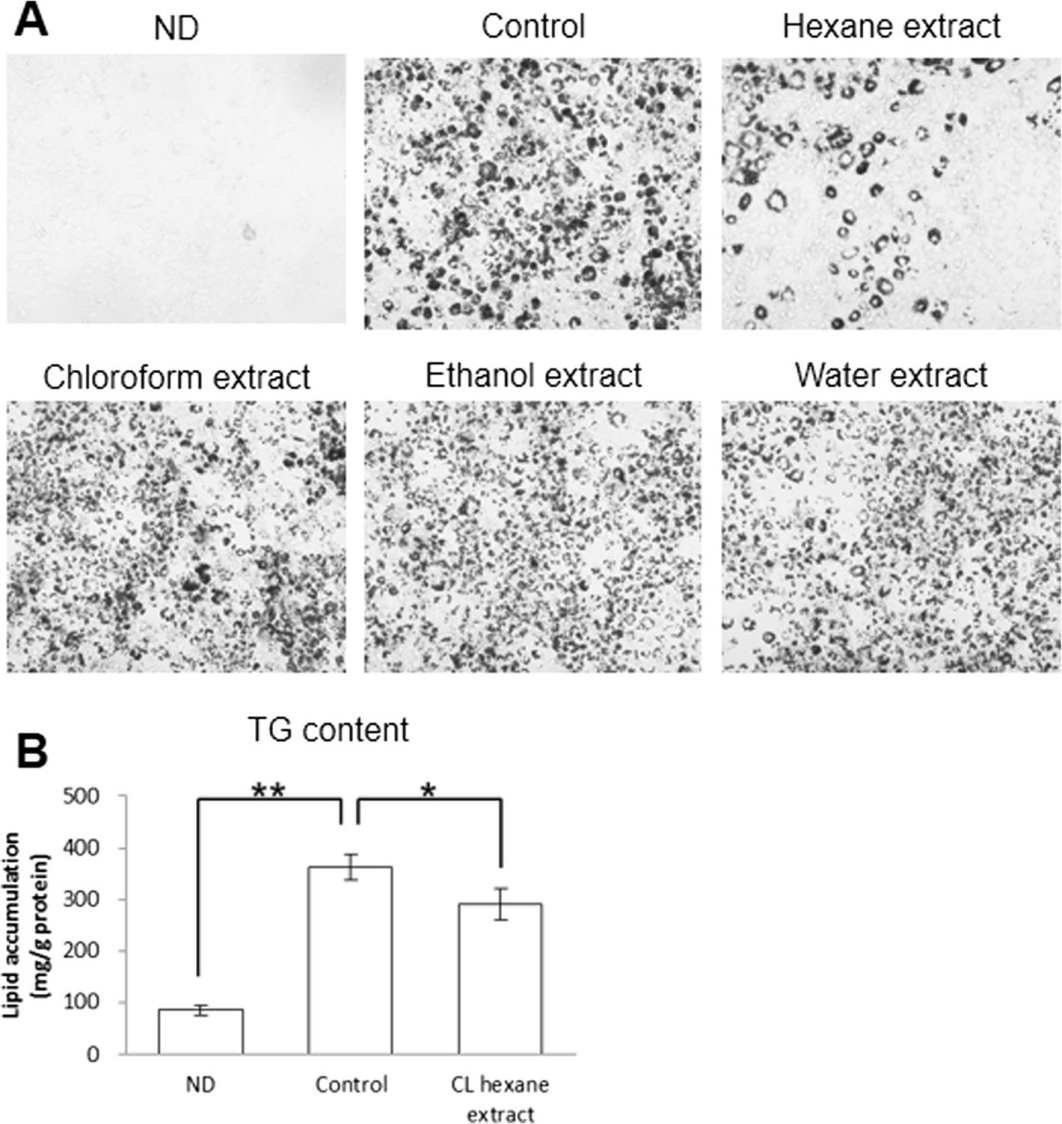
**Effects of *Cirsium brevicaule *A. GRAY leaf (CL) extracts on lipid accumulation in 3T3-L1 adipocytes. (A)** Oil red O staining in 3T3-L1 cells treated with each CL extract. The data from one representative experiment out of three are shown. **(B)** Intracellular triglyceride (TG) content of the 3T3-L1 cells. Each value represents as the means ± SEM for three independent experiments. ND, non-differentiated cells. The asterisks shows significant differences between the experimental groups, determined using Student’s t-test (*: *P* < 0.05, **: *P* < 0.01).

The antiadipogenic activities of serial extracts from CL were tested *in vitro* by using Oil Red O staining (Figure 1A). Red-stained lipid droplets in differentiated adipocytes were not observed in the non-differentiated 3T3-L1 cells (Figure 1A). Adipogenic differentiation of the 3T3-L1 cells significantly increased their lipid content (Figures 1A and 1B). No significant effect on the lipid content of 3T3-L adipocytes was noted with the chloroform, ethanol, or water extracts of CL powder (Additional file 1: Table S2). However, lipid droplet formation was significantly less in the cells treated with the CL hexane extract than in the control cells. The CL extracts did not affect the cell viability of the differentiated 3T3-L1 adipocytes (data not shown).

### Effect of CL hexane extracts on gene expression in 3T3-L1 adipocytes

**Figure 2:**
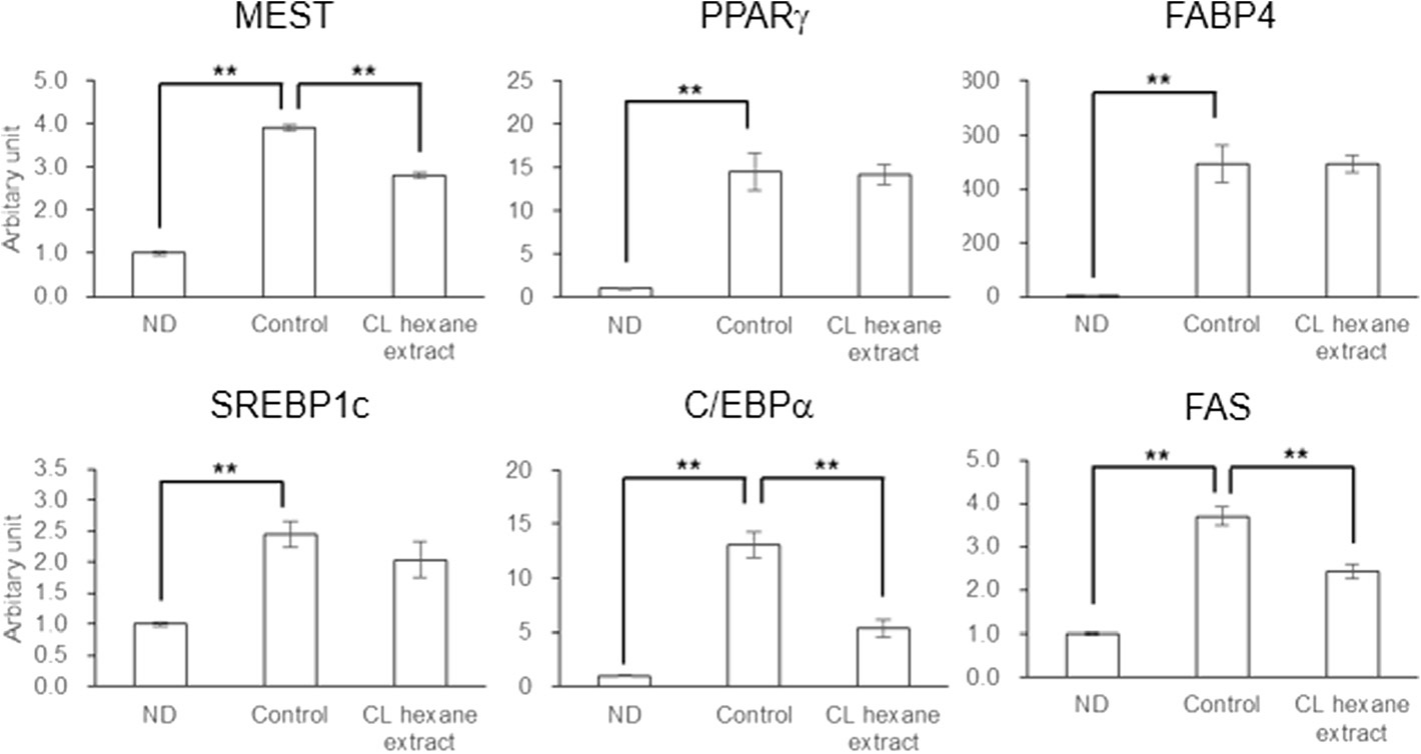
**Effect of *Cirsium brevicaule *A. GRAY leaf (CL) hexane extract on gene expression in 3T3-L1 cells.** Each value represents the means ± S.E.M. for three independent experiments. ND, non-differentiated cells. The asterisks shows significant differences between the experimental groups, determined using Student’s t-test (*: *P* < 0.05, **: *P* < 0.01).

To gain more insight into the effects of the CL hexane extract on lipid metabolism, we examined the gene expression of 3T3-L1 adipocytes that were harvested on day 4 of differentiation. The expressions of all of genes that were determined in this study were enhanced by the induction of adipogenesis (Figure 2). Of these, increases in MEST, C/EBPα and FASN were significantly inhibited by the CL hexane extract.

### Effect of dietary CL on growth, serum, and liver parameters of HFD-fed mice

**Figure 3:**
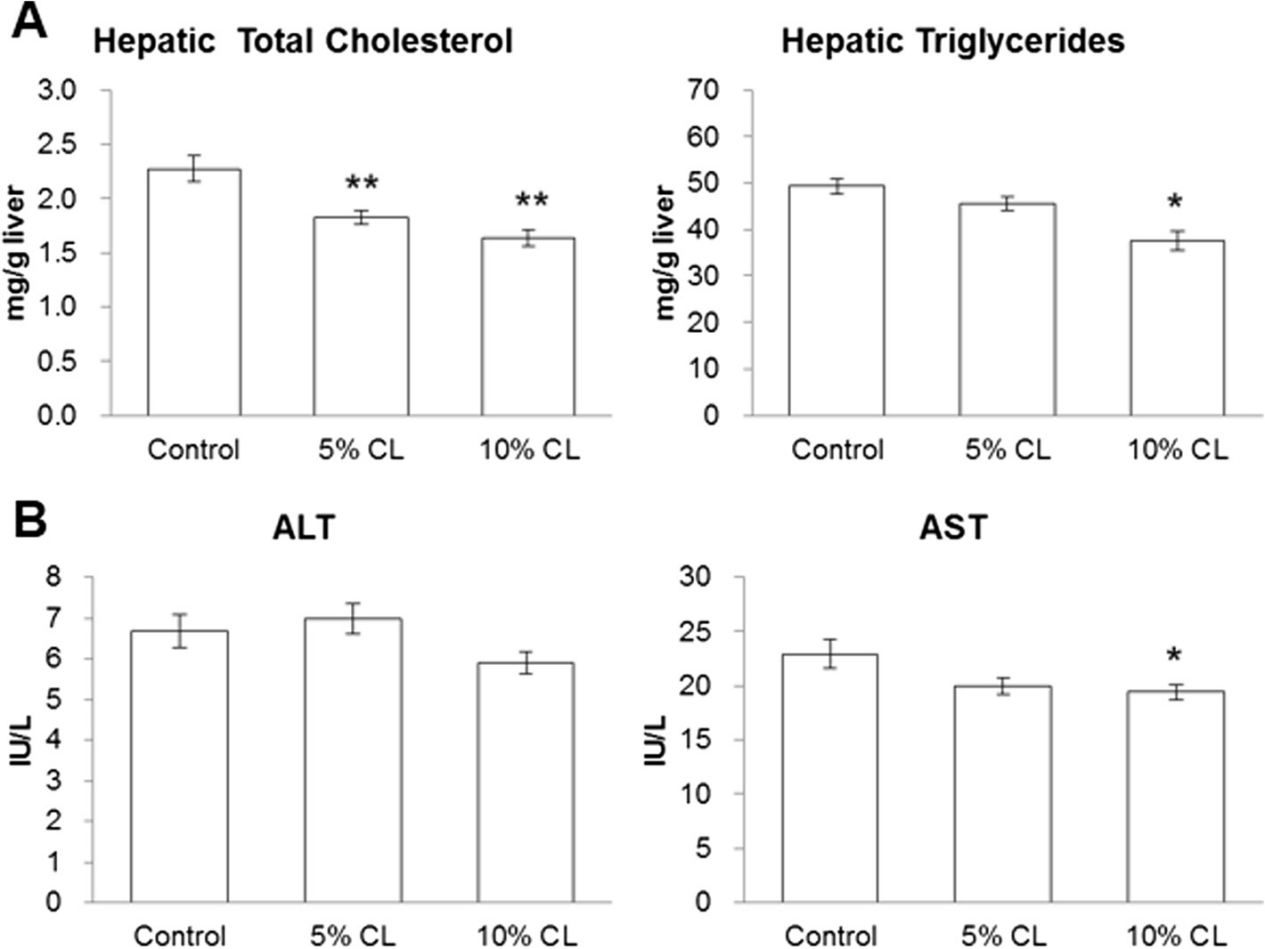
**Effect of dietary *Cirsium brevicaule *A. GRAY leaf (CL) on hepatic physiological parameters in mice. (A)** Serum lipid levels. **(B)** Serum levels of hepatopathic indicator. Each value represents the means ± S.E.M. for six mice. The asterisk shows significant differences when compared to the control group, determined using Dunnett’s test (*: *P* < 0.05, **: *P* < 0.01).

Although food intake and final body weight were largely comparable among the experimental groups, the relative liver weights of 5% or 10% CL for the diet group were significantly lower than those of control diet group (Table 2). The relative weight of subcutaneous WAT was significantly decreased and that of perirenal WAT tended to decrease in the 10% CL diet group compared to the control group. No significant changes were observed in serum levels of TG, TC, glucose, insulin and adiponectin. Serum NEFA levels in the 10% CL diet group were significantly lower than that in the control diet group. In accordance with the decrease in relative liver weight (Table 2), the hepatic TC levels of the CL diet groups were significantly lower than that of control diet group (Figure 3A). The hepatic TG level was also significantly decreased by 10% CL diet. A significant decrease in serum AST activity and a decreasing tendency in serum ALT activity were observed for the 10% CL diet group (Figure 3B).

**Table 2:** Effect of dietary CBAG leaf on growth and serum parameters in mice

**Parameters**	**Group**		
	**Control diet**	**5% CL diet**	**10% CL diet**
Food Intake (g/day)	2.25 ± 0.01	2.22 ± 0.04	2.24 ± 0.03
Initial body weight (g)	13.9 ± 0.32	13.7 ± 0.27	13.7 ± 0.28
Final body weight (g)	25.7 ± 0.43	24.8 ± 0.54	24.5 ± 0.40
Liver weight (g/100 g body weight)	4.50 ± 0.11	3.88 ± 0.17^*^	3.67 ± 0.12^**^
White adipose tissue weight (g/100 g body weight)			
Total^†^	6.03 ± 0.47	5.75 ± 0.36	5.26 ± 0.28
Abdominal^§^	4.34 ± 0.35	4.36 ± 0.31	4.00 ± 0.22
Epididymal	2.18 ± 0.16	2.13 ± 0.16	1.98 ± 0.11
Perirenal	0.81 ± 0.14	0.80 ± 0.07	0.64 ± 0.06
Omental	1.35 ± 0.08	1.44 ± 0.10	1.37 ± 0.08
Subcutaneous	1.69 ± 0.12	1.39 ± 0.06	1.26 ± 0.08**
Serum parameters			
Triglyceride (mg/dL)	35.0 ± 3.41	40.5 ± 3.71	32.2 ± 3.10
Total cholesterol (mg/dL)	107 ± 4	107 ± 4	114 ± 3
Non-esterified fatty acid (mEq/dL)	9.68 ± 0.89	8.41 ± 0.40	6.56 ± 0.56**
Glucose (mg/dL)	210 ± 11	242 ± 11	211 ± 25
Insulin (ng/mL)	1.70 ± 0.23	1.74 ± 0.18	1.79 ± 0.31
Adiponectin (ng/mL)	3.58 ± 0.11	3.51 ± 0.15	3.30 ± 0.13

Each value represents as the means ± S.E.M. for six mice. ^†^Total white adipose tissue weight represents the sum of abdominal and subcutaneous white adipose tissue weights. ^§^Abdominal white adipose tissue weight represents the sum of epididymal, perirenal and omental white adipose tissue weights. *Asterisk* shows significant differences when compared to the control group by Dunnett’s test (*: *P* < 0.05, **: *P* < 0.01).

### Effect of dietary CL on gene expression in the WATs of HFD-fed mice

**Figure 4:**
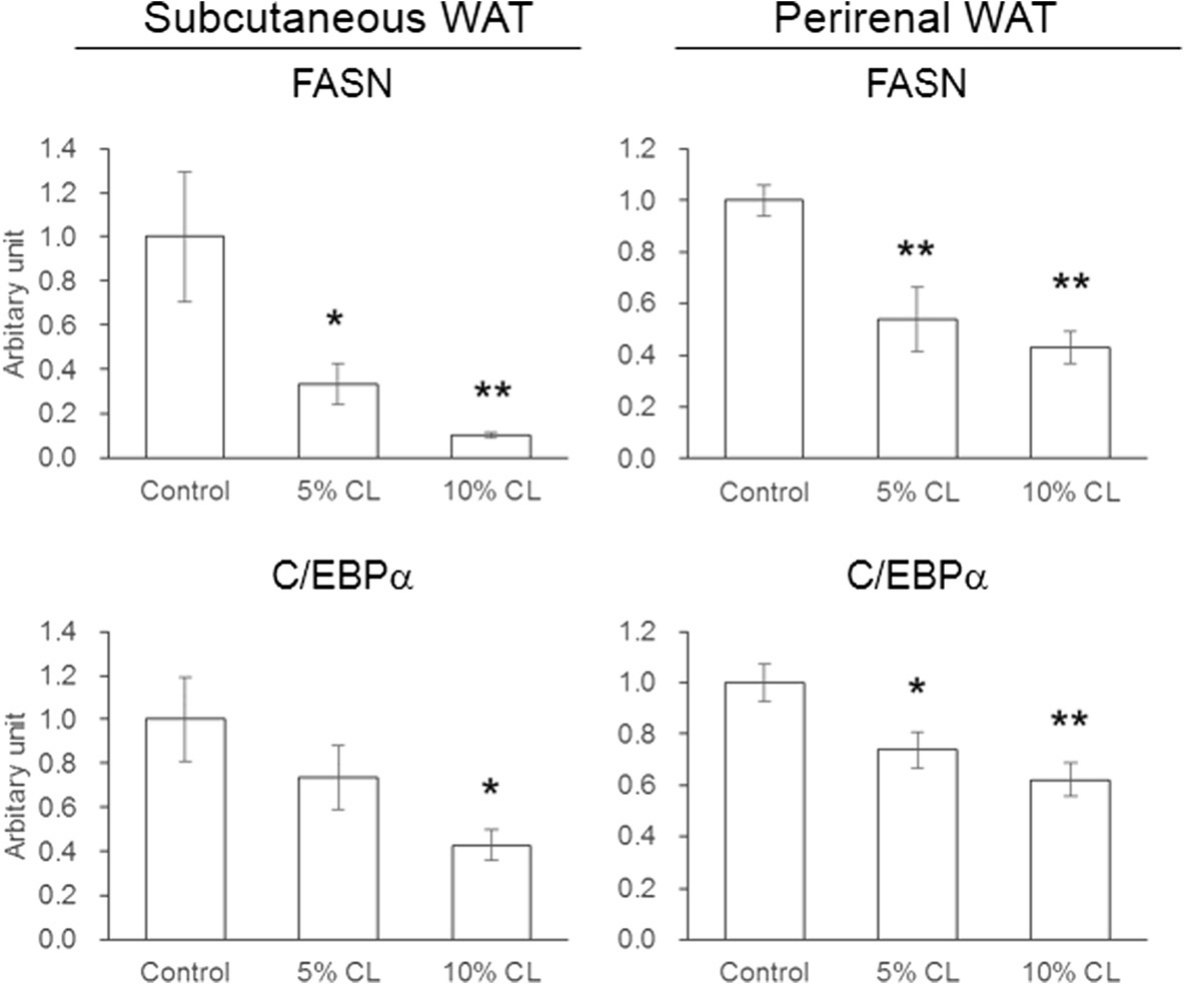
**Effect of dietary *Cirsium brevicaule *A. GRAY leaf (CL) on gene expression in the white adipose tissues of mice.** Each value represents the means ± S.E.M. for six mice. The asterisk shows significant differences when compared to the control group, determined using Dunnett’s test (*: *P* < 0.05, **: *P* < 0.01).

In subcutaneous and perirenal WAT, expression of the FASN gene was significantly reduced by both 5% and 10% CL diets (Figure 4). Similarly, the C/EBPα mRNA levels were decreased by CL diets.

### Effect of dietary CL on gene expression in the livers of HFD-fed mice

**Figure 5:**
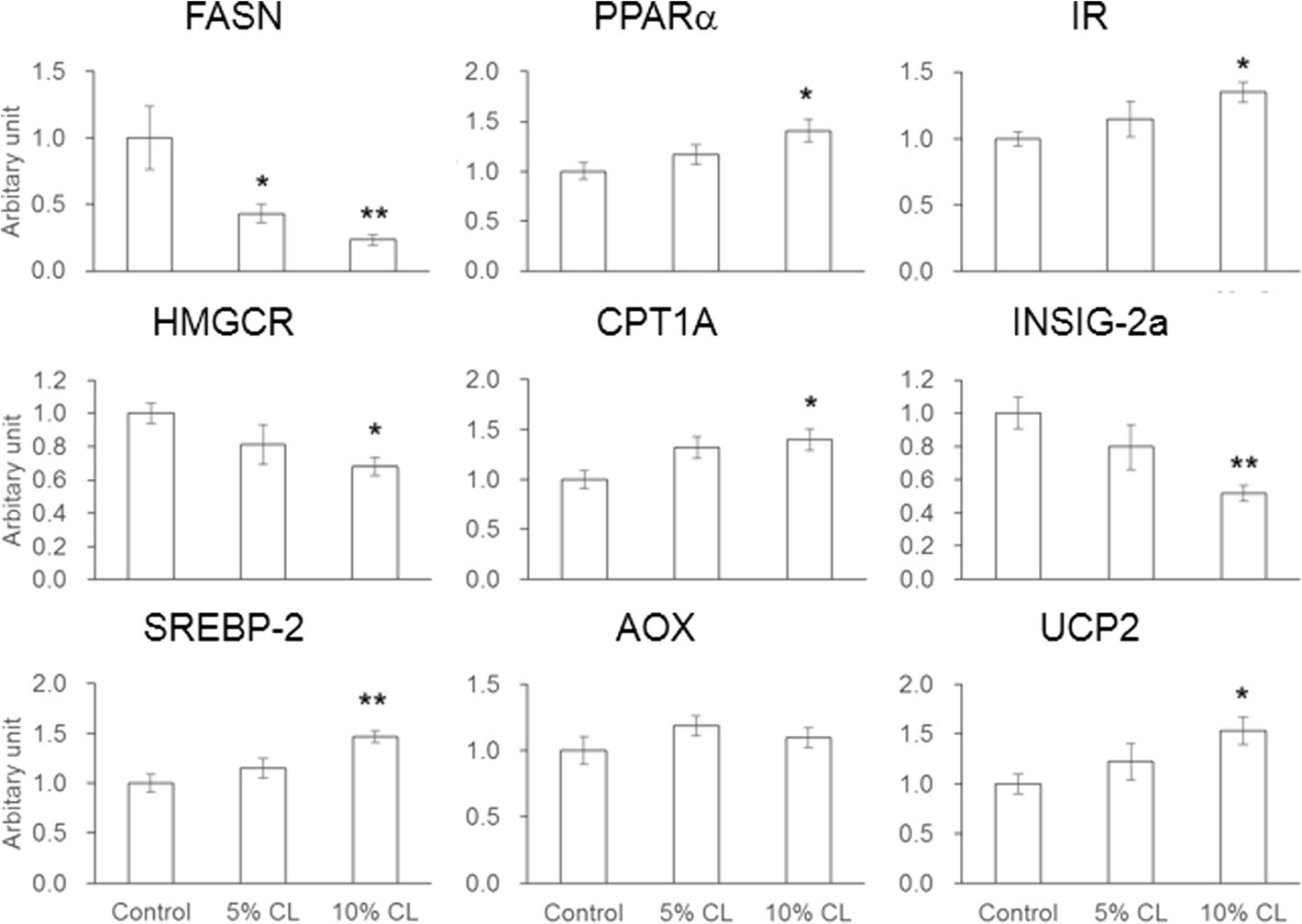
**Effect of dietary CL *Cirsium brevicaule *A. GRAY leaf (CL) on the gene expression in liver of mice.** Each value represents the means ± S.E.M. for six mice. The asterisk shows significant differences when compared to the control group, determined using Dunnett’s test (*: *P* < 0.05, **: *P* < 0.01).

Hepatic mRNA levels of the FASN gene were significantly decreased in the CL diet groups (Figure 5). Significant decreases in HMGCR and INSIG-2a expressions were also observed in the livers of mice fed a 10% CL diet. In contrast, mRNA levels of CPT1A, UCP2, IR, and SREBP-2 were significantly increased by the 10% CL diet. AOX gene expression was largely comparable among the experimental groups.

## Discussion

We investigated for the first time the effects of CL extracts on adipogenesis in adipocytes, and on the development of obesity in B6 mice that are fed a HFD. The CL hexane extract inhibited lipid accumulation and downregulated FASN expression in 3T3-L1 cells (Figures 1 and 2). Dietary CL also suppressed lipid accumulation as well as FASN mRNA levels in subcutaneous WAT and the livers of mice fed a HFD (Table 2, Figures 4 and 5). Dietary CL, on the other hand, increased in the hepatic mRNA levels of genes related to β-oxidation. These results suggest that CL exerts the antiobesity effects partly through downregulation of fatty acid biosynthesis and enhancement of lipolysis.

FASN is highly expressed in adipose tissue and the liver. This enzyme activity is mostly regulated at the transcriptional level [21]. It has been demonstrated that FASN inhibitors such as C75 significantly suppress the differentiation and lipid accumulation in 3T3-L1 cells, which suggests that FASN plays an essential role supporting the differentiation of preadipocytes [17]. Transcription factors such as SREBP-1c, C/EBPα, and PPARγ are known to control the FASN expression [22,23]. In this study, the treatment of 3T3-L1 cells with CL hexane extract significantly decreased their TG content (Figure 1) and MEST gene expression (Figure 2), which could be a novel marker for adipocyte size [24]. Significant decreases in C/EBPα and FASN gene expressions, which were observed with the CL hexane treatment were not necessarily associated with changes in the mRNA levels of SREBP-1c as well as PPARγ and its target gene FABP4. Dietary CL induced a significant reduction or caused a decreasing trend in the WAT weight of the HFD-fed mice (Table 2). Furthermore, the expressions of FASN and C/EBPα in WATs were significantly decreased by the CL diet (Figure 4). These results suggest that the chemical component in CL suppressed adipogenic differentiation or lipid accumulation in adipocytes through the inhibition of FASN gene expression via the downregulation of C/EBPα.

In humans, since exogenous saturated fatty acids are available abundantly through the daily diet, it was considered that *de novo* synthesis of fatty acids was negligible [25]. However, liver-selective FASN inhibition by platensimycin could lead to improved hepatic steatosis and T2DM in model mice [26]. This observation provided the pharmacological proof of concept of FASN inhibition as a unique treatment for obesity-related disorders. In this study, significant decreases in FASN expression in WATs and the liver were observed in the CL-fed mice (Figures 3 and 5). PPARα is well known as an indispensable regulator of liver lipid metabolism. The key enzymes involved in fatty acid oxidation such as CPT1A and AOX are mainly regulated by PPARα [27,28]. Furthermore, PPARα induces UCP2 upregulation, which is involved in the regulation of energy expenditure [29]. The 10% CL diet significantly increased the mRNA levels of PPARα, CPT1A and UCP2, but not AOX (Figure 5). Therefore, these results suggest that dietary CL suppressed hepatic TG content through inhibition of FASN expression and enhancement of fatty acid oxidation, thereby leading to significant reductions in serum NEFA levels in the HFD-fed mice (Table 2). Plasma NEFA levels are elevated in obesity, and a high level of NEFA is considered a critical factor that triggers the onset of peripheral and hepatic insulin resistance [30], eventually leading to development of metabolic syndrome including T2DM and NAFLD. NEFA were recently highlighted to play primary roles in the pathogenesis of NAFLD by inducing inflammation, oxidative stress, apoptosis, and cytokine production in hepatocytes [31].

IR is as known as one of the most important molecules involved in NEFA-induced insulin resistance, because fatty acids suppress the protein and mRNA levels of IR in muscle cells and hepatic cells [32,33]. Although serum insulin levels were largely comparable among the experimental groups in the present study (Table 2), IR gene expression was significantly increased in the liver of mice fed the 10% CL diet (Figure 5). Yabe *et al*. [34] discovered a liver-specific transcript of INSIG-2, designated INSIG-2a, which is downregulated by insulin. Our study showed that the hepatic mRNA level of INSIG-2a in the 10% CL diet group was significantly lower than that in control diet group (Figure 5). Thus, dietary CL likely functions to restore hepatic insulin sensitivities in HFD-fed mice, preventing the development of NAFLD. In contrast, Insigs is known to negatively regulate HMGCR transcription by suppressing the activation of SREBPs [35]. SREBP-2 promotes the mRNA transcription of cholesterol biosynthesis-related genes including HMGCR [36]. A significant increase in SREBP-2 gene expression was herein observed in the livers of the 10% CL diet group (Figure 5), although the mRNA level of HMGCR was significantly decreased. These conflicting results need further clarification to fully understand the anti-obesity activity of CL.

Few studies examined the phenolic compounds in CBAG, although various phenolic compounds have been isolated from the genus *Cirsium*. Pectolinarin have been reported as a major flavonoid in Japanese *Cirsium* species [37], and it was isolated from CBAG [38]. Apigenin, linarin, and luteorin were also isolated from a wide variety of *Cirsium* species [39]. Treatment of 3T3-L1 cells with pectolinarin were significantly increased cellular TG content [11]. Apigenin, but not luteorin, significantly inhibited adipogenesis in 3T3-L1 cells [40]. Although many phenolic compounds have been reported to inhibit lipid accumulation in 3T3-L1 cells [41], the hexane extract of the lowest phenolic content among CL extracts herein inhibited lipid accumulation in 3T3-L1 cells (Figure 1B and Additional file 1: Table S2). This finding suggests that the anti-obesity activity of CL is associated with more hydrophobic non-phenolic compounds. Further purification and identification of active component(s) in CL would be necessary.

Liao *et al.*[11,12] reported that pectolinarin and DDMF, which are flavonoids isolated from *C. japonicum* DC, enhance adipogenic differentiation of 3T3-L1 cells and alleviate streptozotocin-induced diabetes through enhanced PPARγ expression and adiponectin production. Crude extracts prepared from *C. oligophyllum* significantly suppressed the lipid accumulation in WATs by upregulating UCP1 and UCP3 expression in WATs and skin, respectively [13]. In our present study, the hexane extract inhibited adipocyte development and TG accumulation in 3T3-L1 cells with no change in PPARγ gene expression (Figures 1B and 2). Dietary CL reduced the WAT weights without any changes in serum adiponectin levels (Table 2). An inhibition of intestinal TG absorption generally decreased blood TG level, and a similar result is observed with cholesterol [42]. In the present study, significant decrease in serum NEFA level were observed but not in TG and cholesterol levels of CL-fed mice (Table 2). Therefore, the reduction in the adipose tissue weight may be explained by the decreased endogenous NEFA supply for adipogenesis and the enhancement of fatty acid oxidation. Significant changes in the mRNA levels of lipid metabolism-related genes and the lipid content in the livers of the CL diet group appear to support this hypothesis. However, these data do not necessarily negate the possibility that CL interfered the lipid absorption, and thereby exerted the anti-obesity activity as was the case in the previous studies. Further studies are needed to address this question.

## Conclusion

The present results clearly demonstrated the presence of antiobesity phytochemicals in CL, thereby providing scientific evidence to promote the development of natural and safe antiobesity agents from CL. The components in CL remains to be identified, and further studies should be conducted to gain insights into the mechanism of action of CL.

## Abbreviations

ALT: Alanine aminotransferase; AST: Aspartate aminotransferase; AOX: Acyl-CoA oxidase; B6: C57BL/6; CBAG: *Cirsium brevicaule* A GRAY; C/EBP: CCAAT/enhancer binding protein; CL: CBAG leaf; CPT: Calnitine palmitoyltrasferase; DDMF: 5, 7-dihydroxy-6, 4’-dimethoxyflavone; FABP: Fatty acid binding protein; FASN: Fatty acid synthase; HFD: High fat diet; INSIG: Insulin induced gene; IR: Insulin receptor; MEST: Mesoderm-specific transcript; NAFLD: Nonalcoholic fatty liver disease; NEFA: Non-esterified fatty acids; PPAR: Peroxisome proliferator-activated receptor; SREBP: Sterol regulatory element-binding protein; TC: Total cholesterol; TG: Triglyceride; T2DM: Type 2 diabetes mellitus; UCP: Uncoupling protein; WAT: White adipose tissue.

## Competing interests

The authors declare that they have no competing interests.

## Authors’ contributions

MI made a substantial contribution to the conception and design of this study by performing the experiments, assembling, analyzing, and interpreting the data and drafting the manuscript. RN, IF, and AI participated in the experimental work and collected, assembled, and analyzed the data. YK contributed to the conception of this study and the preparation of the materials for the experiments. HO contributed to planning the experiments, discussing the results, and preparing the manuscript. All of the authors have read and approved of the final manuscript.

## Supplementary Material

Additional file 1: Table S1 Primer sequences for real time PCR amplification. **Table S2.** Effects of *Cirsium brevicaule* A. GRAY leaf (CL) extracts on lipid accumulation in 3T3-L1 adipocytes. Click here for file
