# Supplementary material for: Cirsium brevicaule A. GRAY leaf inhibits adipogenesis in 3T3-L1 cells and C57BL/6 mice
Source: Lipids Health Dis. 2013 Aug 15;12:124. doi: 10.1186/1476-511X-12-124 (PMC3751477; doi:10.1186/1476-511X-12-124)
Supplement: Additional file 1: Table S1 — Primer sequences for real time PCR amplification. Table S2. Effects of Cirsium brevicaule A. GRAY leaf (CL) extracts on lipid accumulation in 3T3-L1 adipocytes. [file 1476-511X-12-124-S1.docx]

Additional file 1: Table S1 Primer sequences for real time PCR amplification

Target* Forward primer Reverse primer

ACTB 5’- CAGAAGGAGATTACTGCTCTGGCT -3’ 5’- GGAGCCACCGATCCACACA -3’

18S rRNA 5’- CGGACAGGATTGACAGATTG -3’ 5’- CAAATCGCTCCACCAACTAA -3’

MEST 5’- GTTTTTCACCTACAAAGGCCTACG -3’ 5’- CACACCGACAGAATCTTGGTAGAA -3’

PPARα 5’- CCTCAGGGTACCACTACGGAGT -3’ 5’- GCCGAATAGTTCGCCGAA -3’

PPARγ 5’- AGGCCGAGAAGGAGAAGCTGTTG -3’ 5’- TGGCCACCTCTTTGCTGTGCTC -3’

SREBP-1c 5’- GGAGCCATGGATTGCACATT -3’ 5’- GCTTCCAGAGAGGAGGCCAG -3’

SREBP-2 5’- TCTTCTATACCTGTAGAGCCATGC -3’ 5’- ACATGGTTAAGTGAAGGATCAGAA -3’

FABP4 5’- AGCATCATAACCCTAGATGG -3’ 5’- CATAACACATTCCACCACCAGC -3’

C/EBPα 5’- TGGACAAGAACAGCAACGAGTAC -3’ 5’- GCAGTTGCCCATGGCCTTGAC -3’

FASN 5’- TGCTCCCAGCTGCAGGC -3’ 5’- GCCCGGTAGCTCTGGGTGTA -3’

IR 5’- CCGAAGATTTCCGAGACCTCAG -3’ 5’- GGATACGGGACCAGTCGATAGTG -3’

HMGCR 5’- AAGGTGGTGAGAGAGGTGTTAAAG -3’ 5’- AATACAGTTTGAACTCCCCACATT -3’

CPT1A 5’- AAAGATCAATCGGACCCTAGACA -3’ 5’- CAGCGAGTAGCGCATAGTCA -3’

INSIG-2a 5’- CCCTCAATGAATGTACTGAAGGATT -3’ 5’- TGTGAAGTGAAGCAGACCAATGT -3’

AOX 5’- TCAACAGCCCAACTGTGACTTCCATTA -3’ 5’- TCAGGTAGCCATTATCCATCTCTTCA -3’

UCP2 5’- CAGGTCACTGTGCCCTTACCAT -3’ 5’- CACTACGTTCCAGGATCCCAAG -3’

*ACTB, β-actin; MEST, mesoderm-specific transcript; PPAR, peroxisome proliferator-activated receptor; SREBP, sterol regulatory element-binding protein; FABP4, fatty acid binding protein 4; C/EBPα, CCAAT/enhancer binding protein α; FASN, fatty acid synthase; IR, insulin receptor; HMGCR, HMG-CoA reductase; CPT1A, calnitine palmitoyltrasferase 1A; INSIG-2a, insulin induced gene 2a; AOX, acyl-CoA oxidase; UCP2, uncoupling protein 2.

Additional file 1: Table S2 Effects of *Cirsium brevicaule* A. GRAY leaf (CL) extracts on lipid accumulation in 3T3-L1 adipocytes

Treatment TG content (mg/g protein)

ND^†^ 42.5 ± 3.8

Control 285 ± 9^§^

CL hexane extract 212 ± 11*

CL chloroform extract 268 ± 7

CL ethanol extract 266 ± 6

CL water extract 280 ± 12

Each value represents as the means ± SEM for three independent experiments. ^†^ND, non-differentiated cells. ^§^Significant difference at *P* < 0.05 when compared to ND group by Student’s t-test. *Significant difference at *P* < 0.05 between the experimental differentiated groups when compared to control group by Dunnett’s test.
